# Diagnosis and management of respiratory viruses in critically ill adult patients: an international survey of knowledge and practice among intensivists

**DOI:** 10.1186/s13613-020-00660-0

**Published:** 2020-04-28

**Authors:** Quentin Philippot, Vincent Labbé, Jérémie Pichon, Michel Djibré, Muriel Fartoukh, Guillaume Voiriot

**Affiliations:** Service de Médecine Intensive Réanimation, Hôpital Tenon, Sorbonne Université, Hôpitaux Universitaires de l’Est Parisien, Assistance Publique - Hôpitaux de Paris, Paris, France

## Abstract

In this survey endorsed by the European Society of Intensive Care Medicine (ESICM), we aimed to describe the practice patterns of intensivists worldwide, regarding their diagnosis and management of respiratory viruses in lower respiratory tract infections. There were 229 respondents from 53 countries, mainly in Europe (78%). Our main findings are that a majority of intensivists (i) searched for respiratory viruses in case of severe community-acquired LRTI in adults, whatever the season and the medical history and clinical presentation; (ii) had access to large-panel respiratory mPCR; (iii) used them as first-line diagnostic test in routine practice; (iv) had some knowledge about the panel of the mPCR that they use, but markedly less about the cost. However, we observed strong heterogeneity regarding how intensivists took into account mPCR results for infection control (confinement measures) and patient care (antiviral treatment and antibiotics management).

To the editor,

The role of respiratory viruses, including influenza and non-influenza viruses, in severe lower respiratory tract infections (LRTI) is of increasing concern. However, the approaches for their diagnosis and management may vary among intensivists given the lack of guidance. We consequently aimed at describing the practice patterns of intensivists worldwide using a 39-question self-administered online questionnaire (Additional file 1: Appendix S1). After approval from Research Committee members of the European Society of Intensive Care Medicine (ESICM), the questionnaire was openly accessible on its website from January 24 to June 4, 2019. There were 229 respondents from 53 countries, mainly in Europe (78%), who worked predominantly in mixed intensive care units (74%) (Additional file 1: Table S1).

In case of an acute respiratory failure suggestive of community-acquired LRTI, 83% of the respondents considered searching for respiratory viruses. This attitude was encouraged in case of pre-existing respiratory condition, immunocompromised status or presence of coryza/rhinorrhea. Interestingly, more than three-quarters of respondents confirmed their attitude even in the absence of influenza-like illness or recent close contact with somebody presenting influenza-like illness.

Nucleic acid amplification tests, such as multiplex polymerase chain reaction (mPCR), were available routinely (95%), and largely used as the first-line test to search for respiratory viruses. The test was available only during the opening hours in half of the cases (56%), with a turnaround time below 2 h in only 11% of cases. Large panels (> 5 viruses) and those including atypical bacteria were available for 70% and 47% of the respondents, respectively. Most of them (92%) had some knowledge of the panel composition, but 53% ignored the mPCR cost. The nasopharyngeal swab was the preferred type of proximal sample (66%). In intubated patients, a proximal sample (27%), a distal sample (45%) or both (29%) were collected. Interestingly, 62% of the respondents considered repeating the mPCR in case of negative results despite a high clinical suspicion of viral LRTI (Table 1 and Additional file 1: Table S2).

**Table 1:** Main results of the survey

Item	*N*	*n*	%
*Clinical scenario: a 60*-*year old patient is admitted from the Emergency Department to your ICU for an acute respiratory failure requiring intubation and mechanical ventilation. You suspect a severe community*-*acquired LRTI*			
Regardless of any additional information about the medical history and clinical and biological presentation, would you consider to search for a respiratory virus?	201		
Certainly yes		95	47
Probably yes		77	38
Probably no		23	12
Certainly no		6	3
Ultimately, you decide to search for a respiratory virus. Therefore, what sort of viral test(s) do you routinely use in this situation?	202		
Nuclear acid amplification test, such as PCR		191	95
Viral antigen		40	20
Viral culture		7	3
Other		2	1
What are the characteristics of the panel of the respiratory multiplex PCR that is used in your institution?	202		
The panel includes < 5 respiratory viruses		48	24
The panel includes > 5 respiratory viruses		122	60
The panel includes atypical bacteria		81	40
The panel includes pyogenes		20	10
The panel includes markers of antimicrobial resistance and quantitative bacterial load		7	3
Respiratory multiplex PCR is not available in the institution		28	14
What is your knowledge about the panel of the respiratory multiplex PCR that is used in your institution?^a^	176		
I perfectly know the panel		59	34
I partially know the panel		103	59
I don’t know the panel		14	8
What is your knowledge about the cost of the respiratory multiplex PCR in your hospital?	201		
Known		38	19
Know an estimation		56	27
Unknown		107	53

The clinical scenario provided in the questionnaire is depicted in italic. Results are expressed as number of respondents to the question (*N*) and number of respondents checking the item (*n*)

Detailed results of the complete survey are available in Additional file 1

*ICU* intensive care unit, *LRTI* lower respiratory tract infection, *PCR* polymerase chain reaction

^a^ 22/201 participants who answer to this question don’t know the turnaround time during opening hours

There were wide variations regarding the putative causal role of non-influenza respiratory viruses in severe community-acquired pneumonia (Additional file 1: Figure S1A), but most respondents (96%) considered bacteria–virus coinfection as a risk factor of severity, in line with previous reports [1, 2]. Only 29% of the respondents systematically applied measures to prevent droplet transmission regardless of the season, whereas 49% did so only during the epidemic Flu season. In case of viral documentation, the application (or continuation) of confinement measures depended on the viral species documented, in parallel with its putative causal role (Additional file 1: Figure S1B). Strikingly, conditions of interruption of confinement measures varied widely among respondents. A majority (65%) interrupted them after a specified period (ranging from 5 to more than 10 days), whereas 24% did so after the complete resolution of both the fever and respiratory symptoms and 11% after a negative result of an additional respiratory mPCR (Additional file 1: Table S3).

In case of severe community-acquired pneumonia with documentation of a non-influenza respiratory virus (i.e., *Respiratory Syncytial Virus*), 77% of the respondents did not consider to prescribe antiviral treatment (i.e., ribavirin) if the patient was non-immunocompromised. Finally, in case of no bacterial documentation despite usual microbiological investigations, regardless of any additional information relating to blood tests, only 30% of respondents declared that the viral documentation encouraged them (certainly of likely) to stop antibiotics early (Additional file 1: Table S4).

This survey has several limitations. First, respondents were mainly from Europe, so caution should be exercised on generalizing our results. Second, the survey response rate was unknown, because the questionnaire was openly accessible on the ESICM website. Therefore, how our cohort of respondents was representative of intensivists worldwide is questionable. In particular, we may suspect that physicians interested in respiratory viruses were more prone to participate. This may have led to an overestimation of mPCR and confinement measures prescription rates. Third, some interesting points were not addressed in our questionnaire, in that we decided to limit to 40 items. For example, it would have been interesting to collect additional data regarding the characteristics of the respondents (gender, years of experience in critical care, proportion of immunocompromised patients in their ICU population). The influence of biomarkers such as procalcitonin on antimicrobial treatments could also have been addressed.

In this survey, we observed that a majority of intensivists (i) searched for respiratory viruses in case of severe community-acquired LRTI in adults, whatever the season, the medical history and clinical presentation; (ii) had access to large-panel respiratory mPCR; (iii) used them as first-line diagnostic test in routine practice; (iv) had some knowledge about the panel of the mPCR that they use, but markedly less about the cost. However, we observed strong heterogeneity regarding how intensivists took into account mPCR results for infection control (confinement measures). Moreover, the impact of the mPCR results on patient care (antiviral treatment and antibiotics management) looked limited. These findings highlight the need for further studies in the field of respiratory viruses, aiming to better integrate nucleic acid amplification tests and their results in the management of severe LRTI in adults.

## Supplementary information

**Additional file 1: Table S1.** Characteristics of respondents. **Table S2.** Diagnosis of respiratory virus-associated lower respiratory tract infection. **Table S3.** Interpretation of diagnosis tests in respiratory virus-associated lower respiratory tract infection. **Table S4.** Management of respiratory virus-associated lower respiratory tract infection. **Figure S1.** (A) Interpretation of mPCR. (B) Measures to prevent droplet transmission. **Appendix S1.** Self-administered questionnaire.

## Data Availability

All data generated and analyzed during the study are included in the published article and can be shared upon request.
