# Supplementary material for: Diagnosis and management of respiratory viruses in critically ill adult patients: an international survey of knowledge and practice among intensivists
Source: Ann Intensive Care. 2020 Apr 28;10:50. doi: 10.1186/s13613-020-00660-0 (PMC7188742; doi:10.1186/s13613-020-00660-0)
Supplement: Supplementary file 1 — Additional file 1: Table S1. Characteristics of respondents. Table S2. Diagnosis of respiratory virus-associated lower respiratory tract infection. Table S3. Interpretation of diagnosis tests in respiratory virus-associated lower respiratory tract infection. Table S4. Management of respiratory virus-associated lower respiratory tract infection. Figure S1. (A) Interpretation of mPCR. (B) Measures to prevent droplet transmission. Appendix S1. Self-administered questionnaire. [file 13613_2020_660_MOESM1_ESM.docx]

**Table S1: Characteristics of respondents**

| **Item** | **N** | **n** | **%** |
| --- | --- | --- | --- |
| **In which country do you work?** | 228 |  |  |
| France |  | 79 | 35 |
| Spain |  | 20 | 9 |
| Portugal |  | 17 | 7 |
| United Kingdom |  | 12 | 5 |
| India |  | 8 | 4 |
| Italy |  | 8 | 4 |
| Greece |  | 7 | 3 |
| Australia |  | 4 | 2 |
| Chile |  | 4 | 2 |
| Denmark |  | 4 | 2 |
| Mexico |  | 3 | 1 |
| Poland |  | 3 | 1 |
| Georgia |  | 3 | 1 |
| Belgium |  | 3 | 1 |
| Ireland |  | 3 | 1 |
| Other |  | 51 | 22 |
| **In which type of hospital are you employed?** | 227 |  |  |
| Teaching hospital |  | 175 | 77 |
| Non-teaching hospital |  | 52 | 23 |
| **Type of ICU** | 226 |  |  |
| Mixed |  | 166 | 74 |
| Medical |  | 47 | 21 |
| Neurological |  | 4 | 2 |
| Surgical |  | 3 | 1 |
| Cardiac |  | 3 | 1 |
| Other |  | 3 | 1 |
| ***Number of beds***  <15  Between 15 and 25  >25 | 226 | 93  88  45 | 41  39  20 |
| **Approximate number of patients treated in your ICU per year?** | 225 |  |  |
| <500 |  | 53 | 24 |
| Between 500 and 1000 |  | 104 | 46 |
| >1000 |  | 68 | 30 |
| **Do you have an intermediate care unit?** | 226 |  |  |
| Yes |  | 125 | 55 |
| No |  | 101 | 45 |
| **What is your primary specialism?** | 226 |  |  |
| Intensivist |  | 84 | 37 |
| Anesthesiology |  | 42 | 18 |
| Internal medicine |  | 37 | 16 |
| Pulmonology |  | 28 | 12 |
| Nephrology |  | 10 | 4 |
| Cardiology |  | 4 | 2 |
| Emergency care |  | 4 | 2 |
| Infectious diseases |  | 4 | 2 |
| Neurology |  | 4 | 2 |
| Hematology |  | 3 | 1 |
| Other |  | 6 | 4 |
| **In which year did you get your qualification/specialty in intensive care medicine?** | 215 |  |  |
|  | 2011 [2003; 2016] | | |

**Abbreviations**: ICU = Intensive Care Unit.

Results are expressed as number of respondents to the question (N) and number of respondents checking the item (n) or median [25^th^ percentile; 75^th^ percentile].

**Table S2: Diagnosis of respiratory virus-associated lower respiratory tract infection**

| **Item** | **N** | **n** | **%** |
| --- | --- | --- | --- |
| *A 60-year old patient is admitted from the Emergency Department to your ICU for an acute respiratory failure requiring intubation and mechanical ventilation. You suspect a severe community-acquired LRTI* | | | |
| **Regardless of any additional information about the medical history and clinical and biological presentation, would you consider to search for a respiratory virus?** | 201 |  |  |
| Certainly yes |  | 95 | 47 |
| Probably yes |  | 77 | 38 |
| Probably no |  | 23 | 12 |
| Certainly no |  | 6 | 3 |
| **Among the following details regarding the medical history, which one(s) may encourage you to search for a respiratory virus?** | 204 |  |  |
| More than 65 years old |  | 96 | 47 |
| Pre-existing respiratory condition |  | 131 | 65 |
| Immunocompromised status |  | 164 | 81 |
| At least one child younger than 5 years in the household |  | 95 | 47 |
| None |  | 17 | 8 |
| **Among the following details regarding the medical history, which one(s) may discourage you to search for a respiratory virus?** | 203 |  |  |
| Absence of influenza-like illness symptoms |  | 51 | 25 |
| Absence of a recent close contact with somebody presenting influenza-like illness |  | 34 | 17 |
| Spring/summer season |  | 100 | 49 |
| None |  | 77 | 38 |
| **Among the following clinical abnormalities, which one(s) may encourage you to search for a respiratory virus?** | 203 |  |  |
| Diffuse crackles |  | 109 | 49 |
| Focal crackles |  | 27 | 12 |
| Wheezing |  | 92 | 41 |
| Cough |  | 109 | 49 |
| Coryza / rhinorrhea |  | 168 | 75 |
| Skin rash |  | 104 | 47 |
| None |  | 15 | 7 |
| **Considering the medical history, clinical data, blood tests, and chest X-Ray/CT-scan, you may be able to make an initial diagnosis quickly. Which one(s) will encourage you to search for a respiratory virus?** | 203 |  |  |
| Community-acquired LRTI |  | 171 | 84 |
| Healthcare-associated LRTI |  | 38 | 19 |
| Cardiogenic pulmonary edema |  | 24 | 12 |
| Severe asthma |  | 115 | 57 |
| Acute exacerbation of COPD |  | 136 | 67 |
| None |  | 11 | 5 |
| **Ultimately, you decide to search for a respiratory virus. Therefore, what sort of viral test(s) do you routinely use in this situation?** | 202 |  |  |
| Nuclear acid amplification test, such as PCR |  | 191 | 95 |
| Viral antigen |  | 40 | 20 |
| Viral culture |  | 7 | 3 |
| Other |  | 2 | 1 |
| **The patient is intubated. What type of respiratory tract sample do you preferentially collect and send to the Lab** | 202 |  |  |
| Proximal sample |  | 54 | 27 |
| Distal sample |  | 89 | 45 |
| Both |  | 59 | 29 |
| **Ultimately, you decide to collect and send a proximal sample.What type of sample do you choose to collect?** | 201 |  |  |
| Nasopharyngeal swab |  | 132 | 66 |
| Nasal wash |  | 12 | 6 |
| Oropharyngeal aspirate |  | 11 | 6 |
| Nasopharyngeal aspirate |  | 30 | 15 |
| Throat swab |  | 16 | 8 |
| **What are the characteristics of the panel of the respiratory multiplex PCR that is used in your institution?** | 202 |  |  |
| The panel includes < 5 respiratory viruses |  | 48 | 24 |
| The panel includes > 5 respiratory viruses |  | 122 | 60 |
| The panel includes atypical bacteria |  | 81 | 40 |
| The panel includes pyogenes |  | 20 | 10 |
| The panel includes markers of antimicrobial resistance and quantitative bacterial load |  | 7 | 3 |
| Respiratory multiplex PCR is not available in the institution |  | 28 | 14 |
| **What is your knowledge about the panel of the respiratory multiplex PCR that is used in your institution?** **^#^** | 176 |  |  |
| I perfectly know the panel |  | 59 | 34 |
| I partially know the panel |  | 103 | 58 |
| I don’t know the panel |  | 14 | 8 |
| **What is the turnaround time (time between the collection of the sample and the return of the results to clinicians) of the respiratory multiplex PCR in your institution, during opening hours (9:00 am - 6:00 pm, Monday – Friday)?** **^§^** | 179 |  |  |
| < 2 hours |  | 20 | 11 |
| 2 to 6 hours |  | 54 | 30 |
| 6 to 24 hours |  | 66 | 37 |
| > 24 hours |  | 39 | 22 |
| **Is the respiratory multiplex PCR available outside opening hours in your center?** | 202 |  |  |
| No |  | 113 | 56 |
| Yes, during night-time from Monday to Friday |  | 42 | 21 |
| Yes, on Saturday |  | 49 | 24 |
| Yes, on Sunday |  | 38 | 19 |
| Unknown |  | 23 | 11 |
| **What is your knowledge about the cost of the respiratory multiplex PCR in your hospital?** | 201 |  |  |
| Known |  | 38 | 19 |
| Know an estimation |  | 56 | 27 |
| Unknow |  | 107 | 53 |

**Abbreviations**: ICU = Intensive Care Unit; LRTI = Lower Respiratory Tract Infection; PCR = Polymerase Chain Reaction.

The clinical scenario provided in the questionnaire is depicted in italic. Results are expressed as number of respondents to the question (N) and number of respondents checking the item (n).

**^#^** 26/202 participants who answer to this question have no respiratory mPCR available in their institution.

**^§^** 22/201 participants who answer to this question don’t know the turnaround time during opening hours.

**Table S3: Interpretation of diagnosis tests in respiratory virus-associated lower respiratory tract infection**

| **Items of the questionnaire** | **N** | **n** | **%** |
| --- | --- | --- | --- |
| 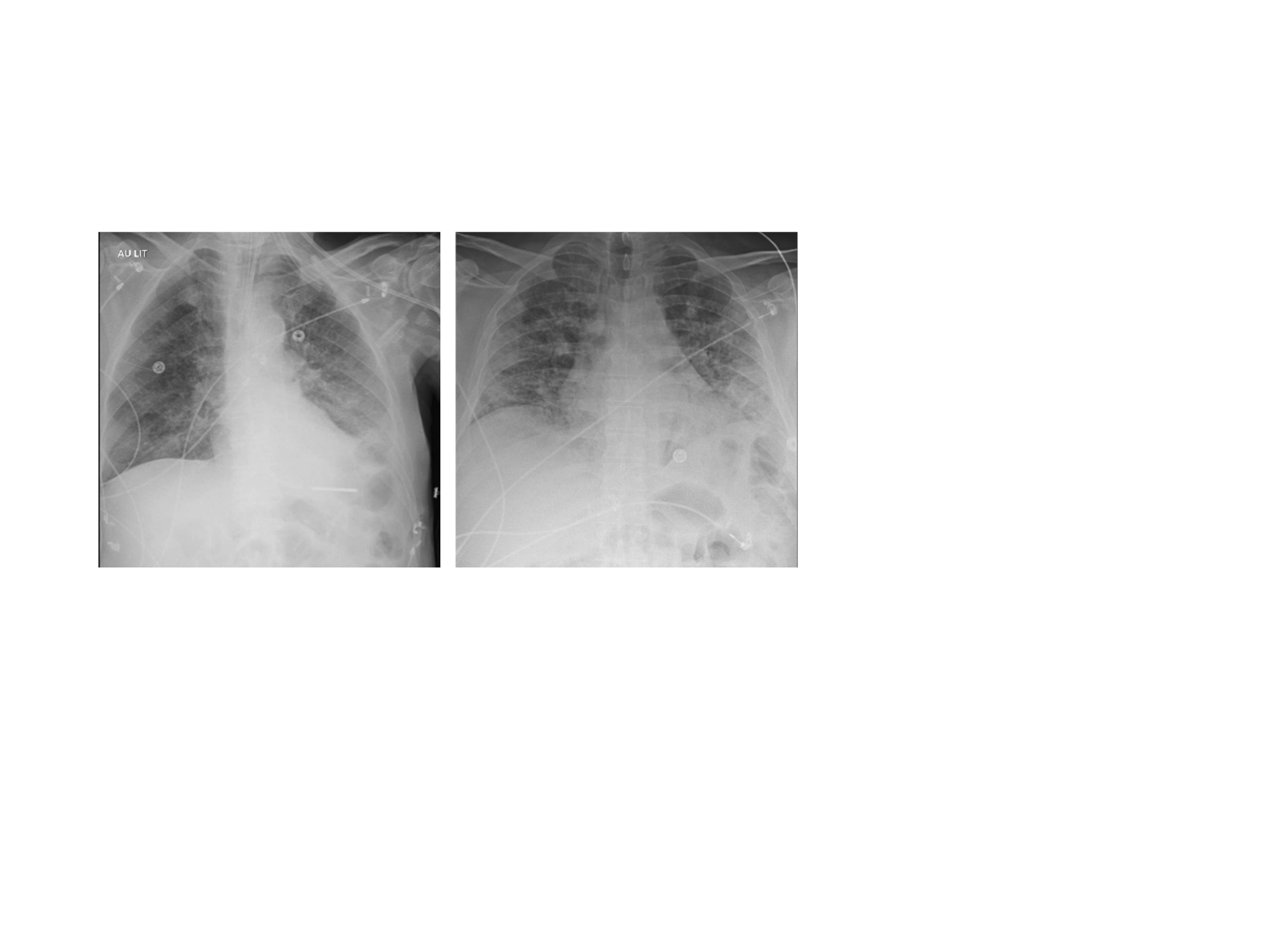*A non-immunocompromised adult patient is admitted from the Emergency Department to your ICU for an acute respiratory failure requiring intubation and mechanical ventilation. You diagnose a community-acquired pneumonia (Figure on the left). All the usual bacteriological investigations are negative, including a tracheal/bronchial aspirate.* | | | |
| **If your suspicion of a viral infection is high but the respiratory multiplex PCR is negative on tracheal/bronchial aspirate, would you consider to repeat the test? ^#^** | 181 |  |  |
| Repeat the test on a new distal sample (tracheal/bronchial aspirate or bronchoalveolar lavage) |  | 81 | 45 |
| Repeat the test on a proximal sample (nasal/nasopharyngeal/throat) |  | 13 | 7 |
| Repeat the test on both distal and proximal samples |  | 19 | 10 |
| Not repeat the test |  | 68 | 38 |
| **Do you consider virus-virus co-infection as a risk factor of severity of LRTI?** | 189 |  |  |
| Certainly |  | 42 | 22 |
| Likely |  | 55 | 29 |
| Possibly |  | 44 | 23 |
| Unlikely |  | 20 | 11 |
| Unknown |  | 28 | 14 |
| **Do you consider bacteria-virus co-infection as a risk factor of severity of LRTI?** | 189 |  |  |
| Certainly |  | 119 | 63 |
| Likely |  | 38 | 20 |
| Possibly |  | 24 | 12 |
| Unlikely |  | 6 | 3 |
| Unknown |  | 2 | 1 |
| **In case of a respiratory virus-associated LRTI, do you consider to repeat the PCR during hospital course to monitor the viral shedding?** | 189 |  |  |
| Always |  | 12 | 6 |
| Often |  | 14 | 7 |
| Sometimes |  | 57 | 30 |
| No |  | 97 | 51 |
| Unknown |  | 9 | 5 |

**Abbreviations**: ICU = Intensive Care Unit; LRTI = Lower Respiratory Tract Infection; PCR = Polymerase Chain Reaction.

The clinical scenario provided in the questionnaire is depicted in italic. Results are expressed as number of respondents to the question (N) and number of respondents checking the item (n).

**^#^** 8/189 participants who answer to this question have no respiratory multiplex available in their institution.

**Table S4: Management of respiratory virus-associated lower respiratory tract infection**

| **Items of the questionnaire** | **N** | **n** | **%** |
| --- | --- | --- | --- |
| *A 60-year old patient is admitted from the Emergency Department to your ICU for an acute respiratory failure, which does not require intubation and mechanical ventilation. You suspect a LRTI of community onset.* | | | |
| **Do you apply measures to prevent droplet transmission?** | 189 |  |  |
| Systematic regardless of the season |  | 55 | 29 |
| Systematic only during the epidemic period |  | 93 | 49 |
| Not applied |  | 39 | 21 |
| Unknown |  | 2 | 1 |
| *A respiratory PCR performed on a proximal sample is positive for one virus.* | | | |
| **What virus(es) will encourage you to apply (or to continue applying) measures to prevent droplet transmission?** | 187 |  |  |
| Rhinovirus |  | 54 | 29 |
| Influenza Virus |  | 176 | 94 |
| Respiratory Syncytial Virus |  | 114 | 61 |
| Human Metapneumovirus |  | 73 | 39 |
| Non-MERS Coronavirus |  | 64 | 34 |
| Parainfluenza Virus |  | 84 | 45 |
| Adenovirus |  | 55 | 29 |
| None |  | 3 | 2 |
| **You decide to apply measures to prevent droplet transmission. When will you interrupt these measures during the stay?** | 187 |  |  |
| After more than 10 days |  | 13 | 7 |
| After 5 to 10 days |  | 52 | 28 |
| After 5 days |  | 57 | 30 |
| After the complete resolution of both the fever and the respiratory symptoms |  | 44 | 24 |
| After you obtain a negative result for a second respiratory PCR |  | 21 | 11 |
| *The virus identified is a Respiratory Syncytial Virus.* | | | |
| **Would you consider to prescribe a specific antiviral treatment, such as Ribavirin?** | 185 |  |  |
| - ***If the patient is non-immunocompromised*** |  |  |  |
| Certainly |  | 7 | 4 |
| Likely |  | 12 | 6 |
| Possibly |  | 20 | 11 |
| Unlikely |  | 142 | 77 |
| Don’t know |  | 4 | 2 |
| - ***If the patient is immunocompromised*** |  |  |  |
| Certainly |  | 46 | 25 |
| Likely |  | 41 | 22 |
| Possibly |  | 60 | 32 |
| Unlikely |  | 35 | 19 |
| Don’t know |  | 3 | 2 |
| 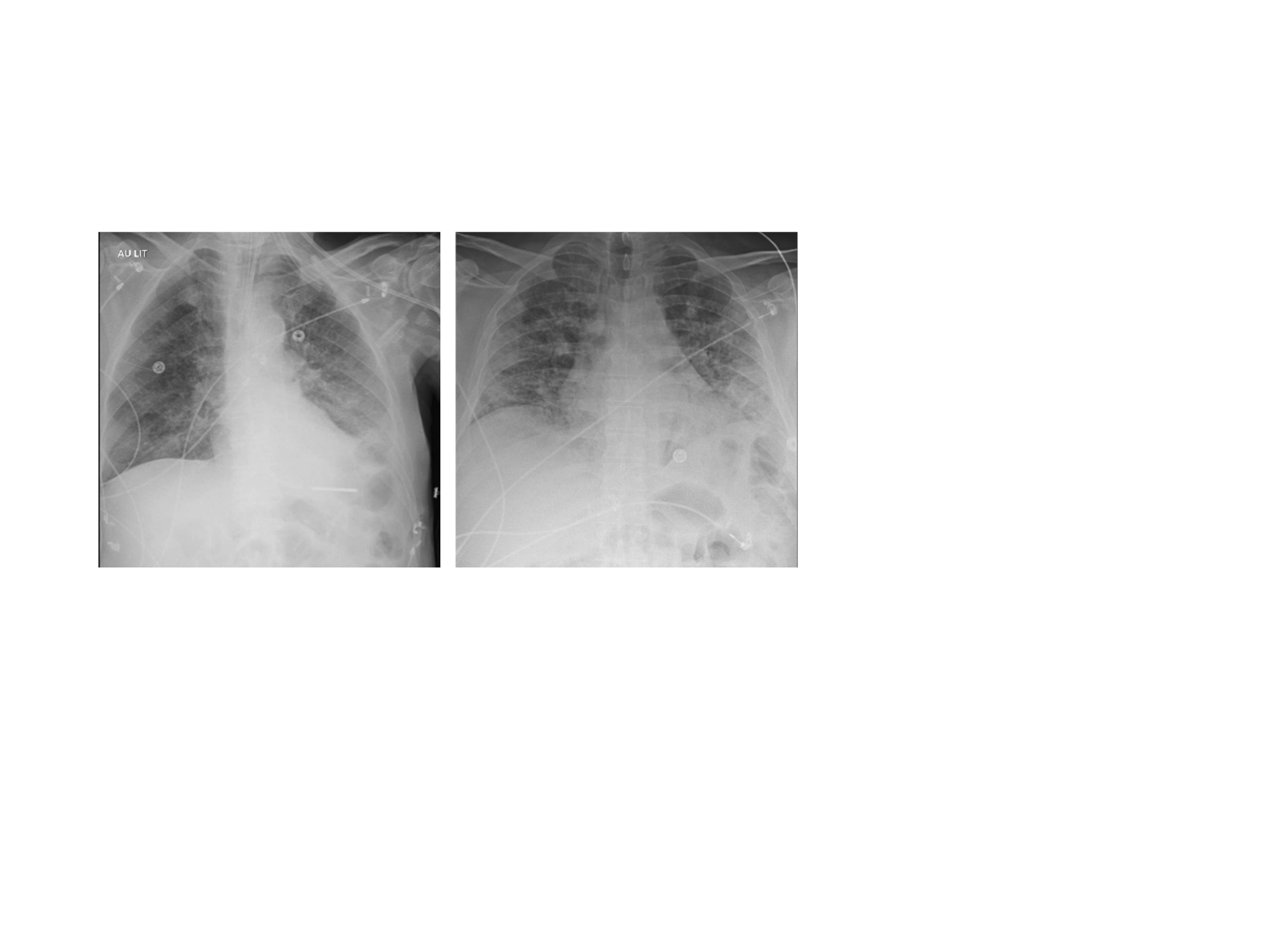*The patient is non-immunocompromised. Chest X-ray reveals progressive bilateral pulmonary infiltrates (Figure on the right). You initiate a combination of antibiotics (cefotaxime plus macrolide). After 48 hours, all the usual bacterial investigations (sputum, blood, urinary antigen tests) are negative.* | | | |
| **Regardless of any additional information related to blood tests and clinical course, would you consider stopping antibiotics right now?** | 185 |  |  |
| Certainly |  | 7 | 4 |
| Likely |  | 9 | 5 |
| Possibly |  | 26 | 14 |
| Unlikely |  | 141 | 76 |
| Don’t know |  | 2 | 1 |
| **Does the documentation of a respiratory virus encourage you to stop antibiotics?** | 185 |  |  |
| Certainly |  | 17 | 9 |
| Likely |  | 38 | 21 |
| Possibly |  | 59 | 32 |
| Unlikely |  | 69 | 37 |
| Don’t know |  | 2 | 1 |

**Abbreviations**: ICU = Intensive Care Unit; LRTI = Lower Respiratory Tract Infections; PCR = Polymerase Chain Reaction.

Results are expressed as number of respondents to the question (N) and number of respondents checking the item (n). The clinical scenario provided in the questionnaire is depicted in italic.

**Figure S1**

***Figure S1A: Interpretation of mPCR***

Context: A non-immunocompromised adult patient is admitted from the Emergency Department to your ICU for an acute respiratory failure requiring intubation and mechanical ventilation. You suspect a community-acquired pneumonia. All the conventional microbiological investigations are negative, including a tracheal/bronchial aspirate.

Question: If a respiratory mPCR is positive for one of these respiratory viruses on tracheal/bronchial aspirate, what would you say regarding the pathogenic role of this virus.

***Figure S1B: Measures to prevent droplet transmission***

Context: A 60-year old patient is admitted from the Emergency Department to your ICU for an acute respiratory failure, which does not require intubation and mechanical ventilation. You suspect a LRTI of community onset.

Question: What virus(es) will encourage you to apply (or to continue applying) measures to prevent droplet transmission?

**Appendix: Self-administered questionnaire**

**Respiratory virus-associated lower respiratory tract infections in critically ill adult patients: a survey of knowledges and practices among European Intensivists.**

**Respiratory Virus-associated LRTI in the ICU**

1. ***In which country do you work?***
2. ***In which city do you work?***
3. ***In which type of hospital are you employed?***

- Teaching hospital
- Non-teaching hospital

1. ***Characteristics of your ICU?***

- Mixed
- Medical
- Surgical
- Cardiac
- Neurological
- Other (please specify):

1. ***Number of beds in your ICU?***

- < 15 beds
- Between 15 and 25 beds
- > 25 beds

1. ***Approximate number of patients treated in your ICU per year?***

- < 500
- 500 to 1000
- > 1000

1. ***Do you have an intermediate care unit (an intermediate care unit is designed to care for patients whose illness is at a level of severity that is intermediate between that which requires ICU facilities and that which can be managed in a conventional ward)?***

- Yes
- No

1. ***What is your primary specialism?***

- Intensive Care
- Anaesthesiology
- Pulmonology
- Cardiology
- Nephrology
- Internal Medicine
- Infectious diseases
- Emergency care
- Other (please specify)

1. ***In which year did you get your qualification/specialty in intensive care medicine?***

***Year of qualification/specialty*: _ _ _ _ _ _ _ _ _ _ _ _ _ _ _ _ _ _ _ _ _ _ _ _ _**

**Diagnosis of respiratory virus-associated severe lower respiratory tract infections**

**A 60-year old patient is admitted from the emergency department to your ICU for an acute respiratory failure requiring intubation and mechanical ventilation. You suspect a severe, community-acquired LRTI.**

***9A. Regardless of any additional information about the medical history and clinical and biological presentation, would you consider to search for a respiratory virus?***

- Certainly no
- Probably no
- Probably yes
- Certainly yes
- I don’t know

***9.B Among the following details regarding the medical history, which one(s) may encourage you to search for a respiratory virus?***

- More than 65-year old
- Pre-existing respiratory condition
- Immunocompromised status
- At least one child younger than 5 years in the household
- None

***9C. Among the following details regarding the medical history, which one(s) may discourage you to search for a respiratory virus?***

- Absence of influenza-like illness symptoms
- Absence of a recent close contact with somebody presenting influenza-like illness
- Spring/summer season
- None

***9D. Among the following clinical abnormalities, which one(s) may encourage you to search for a respiratory virus?***

- Diffuse crackles
- Focal crackles
- Wheezing
- Cough
- Coryza/rhinorrhoea
- Skin rash
- None

***9E. Considering the medical history, clinical data, blood tests, and chest X-Ray/CT-scan, you may be able to make an initial diagnosis quickly. Which one(s) will encourage you to search for a respiratory virus?***

- Community-acquired LRTI
- Healthcare-associated LRTI
- Cardiogenic pulmonary edema
- Severe asthma
- Acute exacerbation of COPD
- None

***9F. Ultimately, you decide to search for a respiratory virus. Therefore, what sort of viral test(s) do you routinely use in this situation?***

- Nuclear acid amplification test, such as Polymerase Chain Reaction (PCR)
- Viral antigen(s)
- Viral culture
- Other

***9G. The patient is intubated. What type of respiratory tract sample do you preferentially collect and send to the lab?***

- A distal sample (tracheal/bronchial aspirate or bronchoalveolar lavage)
- A proximal sample (nasal/nasopharyngeal/oropharyngeal)
- Both distal and proximal samples
- None

***9H. Ultimately, you decide to collect and send a proximal sample. Therefore, what type of sample do you choose to collect?***

- Nasopharyngeal swab
- Nasal wash
- Nasopharyngeal aspirate
- Oropharyngeal aspirate
- Throat swab

1. ***What are the characteristics of the panel of the respiratory multiplex PCR that is used in your institution?***

- The panel includes < 5 respiratory viruses
- The panel includes > 5 respiratory viruses
- The panel includes atypical bacteria
- The panel includes pyogenes
- The panel includes markers of antimicrobial resistance and quantitative bacterial load
- Respiratory multiplex PCR is not available in my institution

1. ***What is your knowledge about the panel of the respiratory multiplex PCR that is used in your institution?***

- I perfectly know the panel
- I partially know the panel
- I don't know the panel
- Respiratory multiplex PCR is not available in my institution

1. ***What is the turnaround time (time between the collection of the sample and the return of the results to clinicians) of the respiratory multiplex PCR in your institution, during opening hours (9:00 am - 6:00 pm, Monday – Friday)?***

- < 2 hours
- 2 to 6 hours
- 6 to 24 hours
- > 24 hours
- I don’t know

1. ***Is the respiratory multiplex PCR available outside opening hours in your center?***

- No
- Yes, during night-time from Monday to Friday
- Yes, on Saturday
- Yes, on Sunday
- I don’t know

1. ***What is your knowledge about the cost of the respiratory multiplex PCR in you hospital?***

- I know the cost
- I can estimate the cost
- I don't know

**Interpretation of diagnosis tests during respiratory virus-associated severe lower respiratory tract infections**


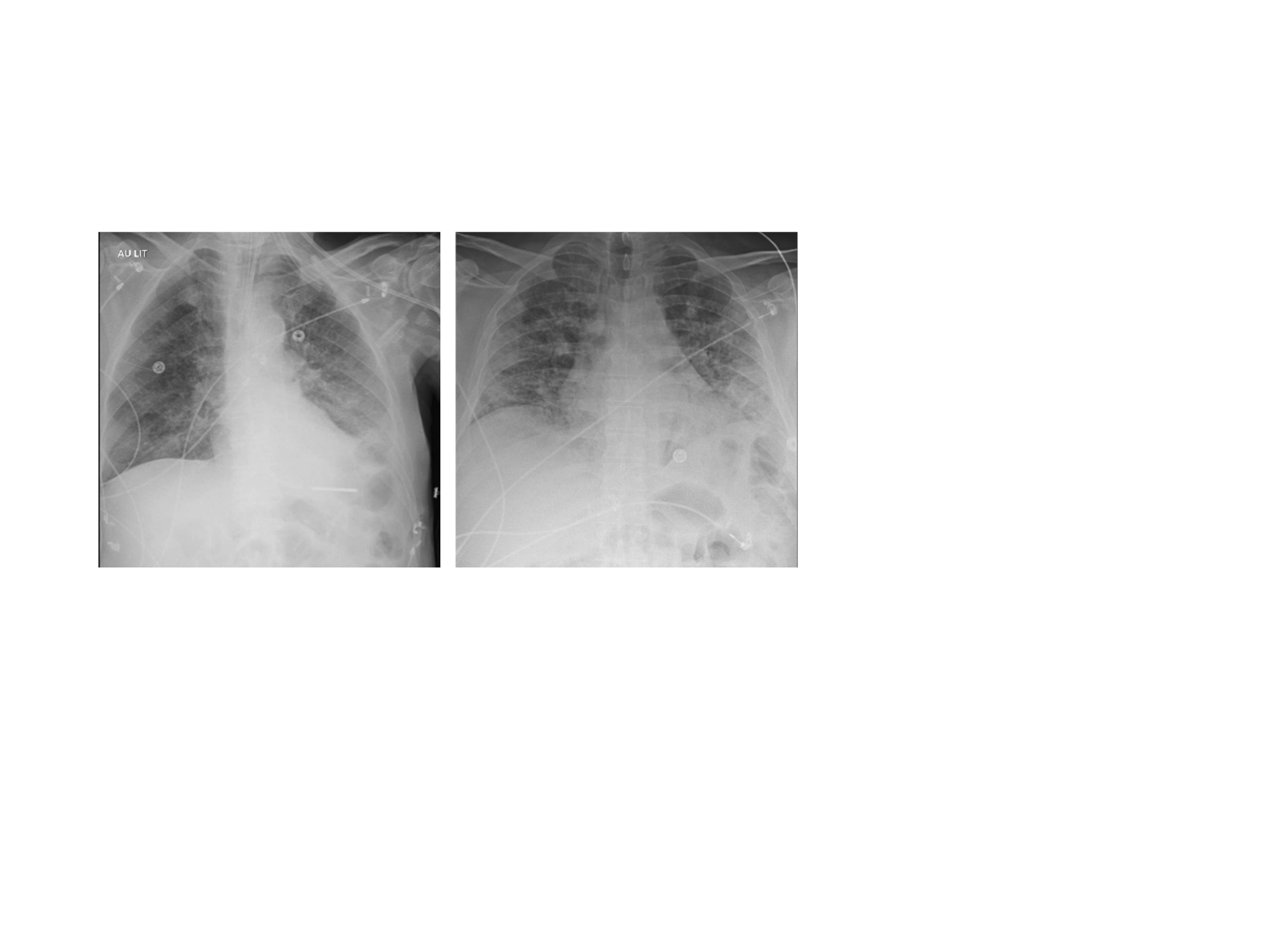


1. **A non-immunocompromised adult patient is admitted from the emergency department to your ICU for an acute respiratory failure requiring intubation and mechanical ventilation. You diagnose a community-acquired pneumonia (Figure on the left). All the usual bacteriological investigations are negative, including a tracheal/bronchial aspirate.**

***15A. If a respiratory multiplex PCR is positive for Human Rhinovirus on tracheal/bronchial aspirate, what would you say regarding the pathogenic role of this virus?***

- Pneumonia is certainly due to this virus
- Pneumonia is likely due to this virus
- Pneumonia is possibly due to this virus
- Pneumonia is unlikely due to this virus
- I don’t know

***15B. Same question with Respiratory Syncitial Virus?***

- Pneumonia is certainly due to this virus
- Pneumonia is likely due to this virus
- Pneumonia is possibly due to this virus
- Pneumonia is unlikely due to this virus
- I don’t know

***15C. Same question with Human Metapneumovirus?***

- Pneumonia is certainly due to this virus
- Pneumonia is likely due to this virus
- Pneumonia is possibly due to this virus
- Pneumonia is unlikely due to this virus
- I don’t know

***15D. Same question with non-MERS Coronavirus (229E, NL63, OC43…)?***

- Pneumonia is certainly due to this virus
- Pneumonia is likely due to this virus
- Pneumonia is possibly due to this virus
- Pneumonia is unlikely due to this virus
- I don’t know

***15E. Same question with Parainfluenza Virus?***

- Pneumonia is certainly due to this virus
- Pneumonia is likely due to this virus
- Pneumonia is possibly due to this virus
- Pneumonia is unlikely due to this virus
- I don’t know

***15F. Same question with Adenovirus?***

- Pneumonia is certainly due to this virus
- Pneumonia is likely due to this virus
- Pneumonia is possibly due to this virus
- Pneumonia is unlikely due to this virus
- I don’t know

***15G. If your suspicion of a viral infection is high but the respiratory multiplex PCR is negative on tracheal/bronchial aspirate, would you consider to repeat the test?***

- No
- Yes, on a new distal sample (tracheal/bronchial aspirate or bronchoalveolar lavage)
- Yes, on a proximal sample (nasal/nasopharyngeal/throat)
- Yes, on both distal and proximal samples
- No respiratory multiplex PCR is available in my institution.

1. ***Do you consider virus-virus co-infection as a risk factor of severity of LRTI?***

- Yes, certainly
- Yes, likely
- Yes, possibly
- No, unlikely
- I don’t know

1. ***Do you consider bacteria-virus co-infection as a risk factor of severity of LRTI?***

- Yes, certainly
- Yes, likely
- Yes, possibly
- No, unlikely
- I don’t know

1. ***In case of a respiratory viral LRTI, do you consider to repeat the PCR during hospital course to monitor the viral shedding?***

- Yes, always
- Yes, often
- Yes, sometimes
- No
- I don’t know

**Management of respiratory virus-associated lower respiratory tract infection**

1. **A 60-year old patient is admitted from the Emergency Department to your ICU for an acute respiratory failure, which does not require intubation and mechanical ventilation. You suspect a LRTI of community onset.**

**19A. *Do you apply measures to prevent droplet*** ***transmission?***

- Yes, regardless of the season
- Yes, exclusively during the Fall/Winter (epidemic) season
- No
- I don’t know

**A respiratory PCR performed on a proximal sample is positive for one virus.**

**19B. *What virus(es) will encourage you to apply (or to continue applying) measures to prevent droplet transmission?***

- Rhinovirus
- Influenza Virus
- Respiratory Syncitial Virus
- Human Metapneumovirus
- Non-MERS Coronavirus (229E, NL63, OC43…)
- Parainfluenza Virus
- Adenovirus
- None

**19C. *You decide to apply measures aiming to prevent droplet transmission. When will you interrupt these measures during the stay?***

- After 5 days
- After 5 to 10 days
- After more than 10 days
- After the complete resolution of both the fever and the respiratory symptoms
- After you obtain a negative result for a second respiratory PCR (no more viral shedding)

**The virus identified is a Respiratory Syncytial Virus.**

***19D. If the patient is non-immunocompromised, would your consider to prescribe a specific antiviral treatment, such as Ribavirin?***

- Yes, certainly
- Yes, likely
- Yes, possibly
- No, unlikely
- I don’t know

***19E. If the patient is immunocompromised, i.e. solid organ transplant, would your consider to prescribe a specific antiviral treatment, such as Ribavirin?***

- Yes, certainly
- Yes, likely
- Yes, possibly
- No, unlikely
-
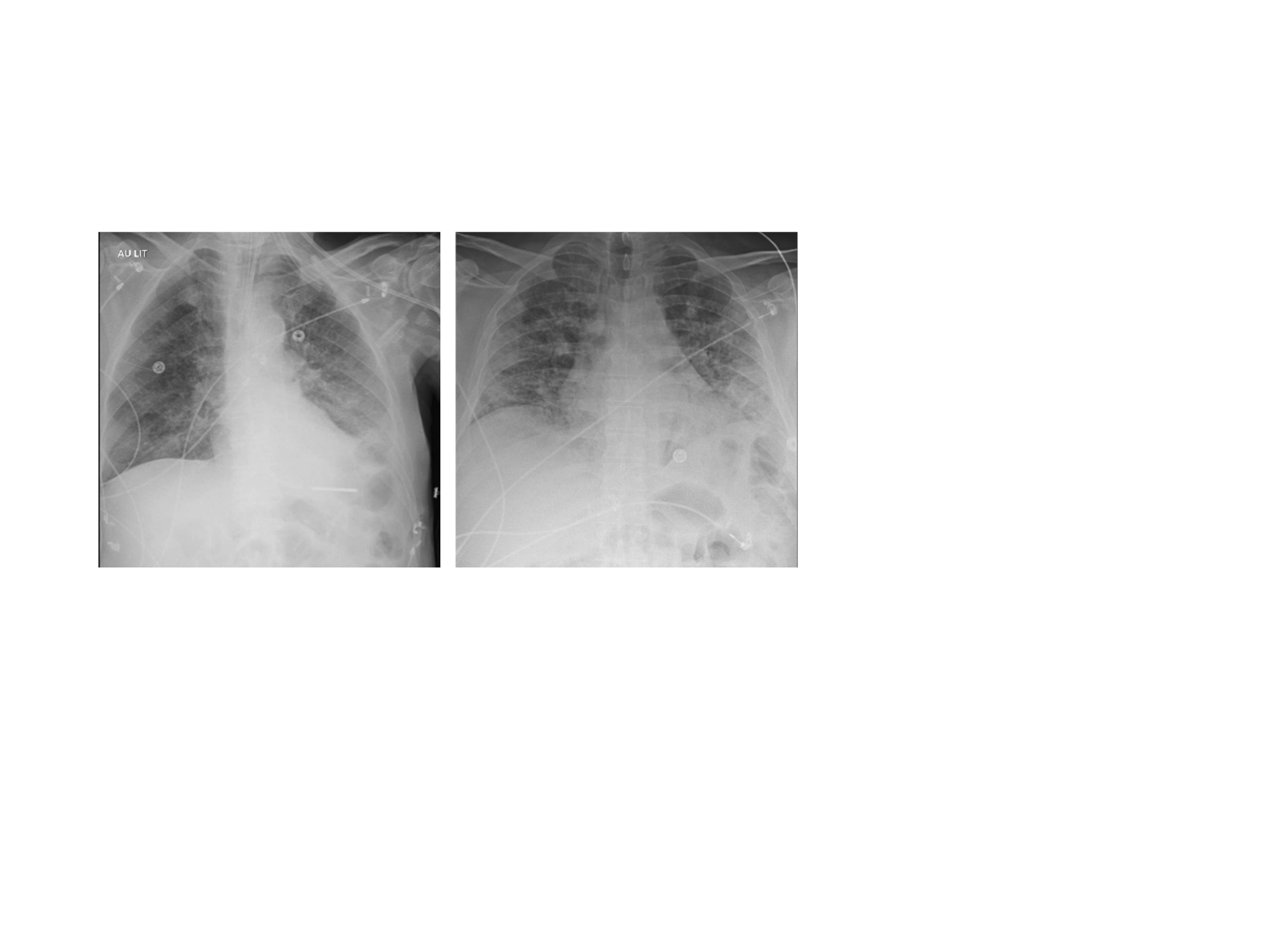
I don’t know

**The patient is non-immunocompromised. Chest X-ray reveals progressive bilateral pulmonary infiltrates (Figure on the right). You initiate a combination of antibiotics (cefotaxime plus macrolide). After 48 hours, all the usual bacterial investigations (sputum, blood, urinary antigen tests) are negative.**

***19F. Regardless of any additional information related to blood tests and clinical course, would you consider stopping antibiotics right now?***

- Yes, certainly
- Yes, likely
- Yes, possibly
- No, unlikely
- I don’t know

***19G. Does the documentation of a respiratory virus encourage you to stop antibiotics?***

- Yes, certainly
- Yes, likely
- Yes, possibly
- No, unlikely
- I don’t know
